# Stable binding to phosphatidylserine-containing membranes requires conserved arginine residues in tandem C domains of blood coagulation factor VIII

**DOI:** 10.3389/fmolb.2022.1040106

**Published:** 2022-10-26

**Authors:** Shaun C. Peters, Kenneth C. Childers, Corbin E. Mitchell, Nathan G. Avery, Steven S. Reese, Cristopher Mitchell, Serena W. Wo, Christopher D. Swanson, Caileen M. Brison, P. Clint Spiegel

**Affiliations:** Department of Chemistry, Western Washington University, Bellingham, WA, United States

**Keywords:** factor VIII, blood coagulation, X-ray crystallography, antibody inhibitors, membrane binding

## Abstract

At sites of vascular damage, factor VIII (fVIII) is proteolytically activated by thrombin and binds to activated platelet surfaces with activated factor IX (fIXa) to form the intrinsic “tenase” complex. Previous structural and mutational studies of fVIII have identified the C1 and C2 domains in binding to negatively charged membrane surfaces through β-hairpin loops with solvent-exposed hydrophobic residues and a ring of positively charged basic residues. Several hemophilia A-associated mutations within the C domains are suggested to disrupt lipid binding, preventing formation of the intrinsic tenase complex. In this study, we devised a novel platform for generating recombinant C1, C2, and C1C2 domain constructs and performed mutagenesis of several charged residues proximal to the putative membrane binding region of each C domain. Binding measurements between phosphatidylserine (PS)-containing lipid membrane surfaces and fVIII C domains demonstrated an ionic strength dependence on membrane binding affinity. Mutations to basic residues adjacent to the surface-exposed hydrophobic regions of C1 and C2 differentially disrupted membrane binding, with abrogation of binding occurring for mutations to conserved arginine residues in the C1 (R2163) and C2 (R2320) domains. Lastly, we determined the X-ray crystal structure of the porcine fVIII C2 domain bound to *o*-phospho-L-serine, the polar headgroup of PS, which binds to a basic cleft and makes charge-charge contact with R2320. We conclude that basic clefts in the fVIII C domains bind to PS-containing membranes through conserved arginine residues *via* a C domain modularity, where each C domain possesses modest electrostatic-dependent affinity and tandem C domains are required for high affinity binding.

## 1 Introduction

Factor VIII (fVIII) is an essential cofactor in the intrinsic pathway of the blood coagulation cascade in which activated fVIII (fVIIIa) associates to phosphatidyl-L-serine (PS) presented on the surface of activated platelet membranes. At the PS membrane surface, fVIIIa binds to activated factor IX (fIXa), forming the active intrinsic “tenase” complex which catalyzes factor X activation (Mann et al., 1990; Gilbert and Drinkwater, 1993). Disruptions to fVIII cofactor activity cause hemophilia A, an X-linked coagulopathy that affects one in 5,000 males and results in life-threating bleeding episodes due to reduction in the proteolytic activity of fIXa (van Dieijen et al., 1981).

**FIGURE 1:**
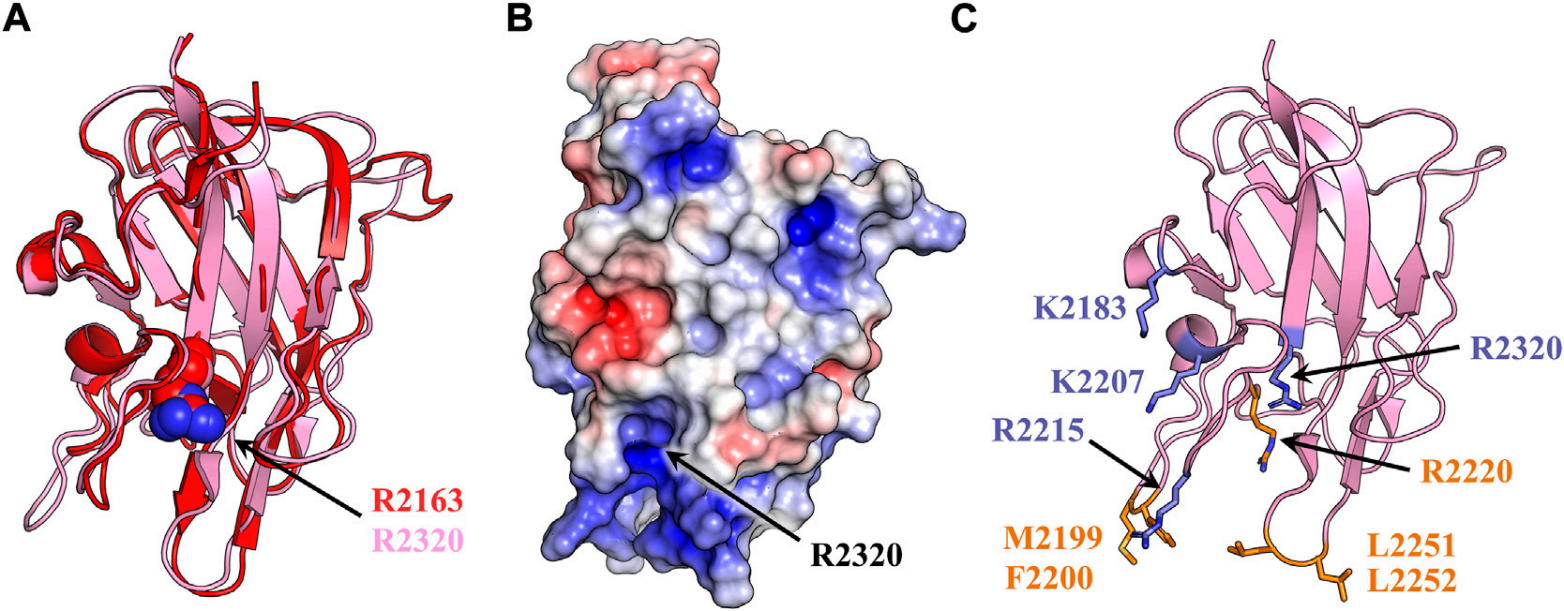
fVIII C2 domain structural homology and membrane binding model. (A) Structural alignment of fVIII C1 (red) and C2 domain (light pink) (PDB: 6MF2). Both domains consist of a homologous β-barrel motif and three solvent exposed hydrophobic loops. Spheres represent a conserved arginine (red: R2163, light pink: R2320). **(B)** Surface electrostatic mapping of the C2 domain. Residue R2320 is located in the most basic cleft of the C2 domain. The surface potential calculation was performed in PyMOL with APBS using a surface potential value of ±5 kT/e (blue: positive charge, red: negative charge, white: neutral). **(C)** Anti-C2 classical antibody inhibitors 3E6 and BO2C11 disrupt fVIII binding to lipid membranes. Sticks represent membrane-binding residues which become buried upon binding to 3E6 (slate) or BO2C11 (orange).

Full-length, mature fVIII is a heterodimer of the heavy chain (A1-A2-B) and light chain (*a3*-A3-C1-C2). Proteolytic activation by thrombin at A1-A2, A2-B, and *a3*-A3 linkers allows activated fVIII (fVIIIa) to dissociate from von Willebrand factor (vWF) and bind to activated platelets with high affinity (1–2 nM) (van Dieijen et al., 1981; Eaton et al., 1986; Hill-Eubanks et al., 1989; Regan and Fay, 1995). Association of fVIIIa to membranes is coordinated by two tandem carboxy-terminal C domains (Figure 1A) (Hsu et al., 2008; Shen et al., 2008; Lu et al., 2011). The C domains of fVIII, termed C1 and C2, are 16 kDa β-sandwich folds that share 66% sequence identity (Pratt et al., 1999; Liu et al., 2000).

Current fVIII membrane binding models rely exclusively on the biochemical interplay between the hydrophobic and electrostatic properties of the C2 domain. High-resolution crystal structures of the C2 domains from factor V (fV) and fVIII have revealed solvent exposed, hydrophobic residues which are implicated in membrane docking by favorably burying hydrophobic residues into the anhydrous bilayer of activated platelets (Macedo-Ribeiro et al., 1999; Pratt et al., 1999). Mutagenesis studies targeting these residues demonstrate that M2199-F2200, L2251-L2252, and W2313-H2315 are required for optimal platelet binding in a PS-dependent manner (Gilbert et al., 2002; Liu et al., 2010). Binding data indicate fVIII contains an isomeric-dependent binding preference to O-phosphatidyl-L-serine (OPLS), suggesting that at least one stereospecific binding site for PS headgroups exists (Gilbert and Drinkwater, 1993). A ring of basic residues positioned above each set of hydrophobic spikes has been implicated in forming favorable charge-charge interactions with negatively charged PS headgroups (Pratt et al., 1999). This region of the C2 domain is highly basic, with electrostatic modeling of the C2 domain structure conveying that the most basic residue within this cleft is R2320 (Figure 1B).

**FIGURE 2:**
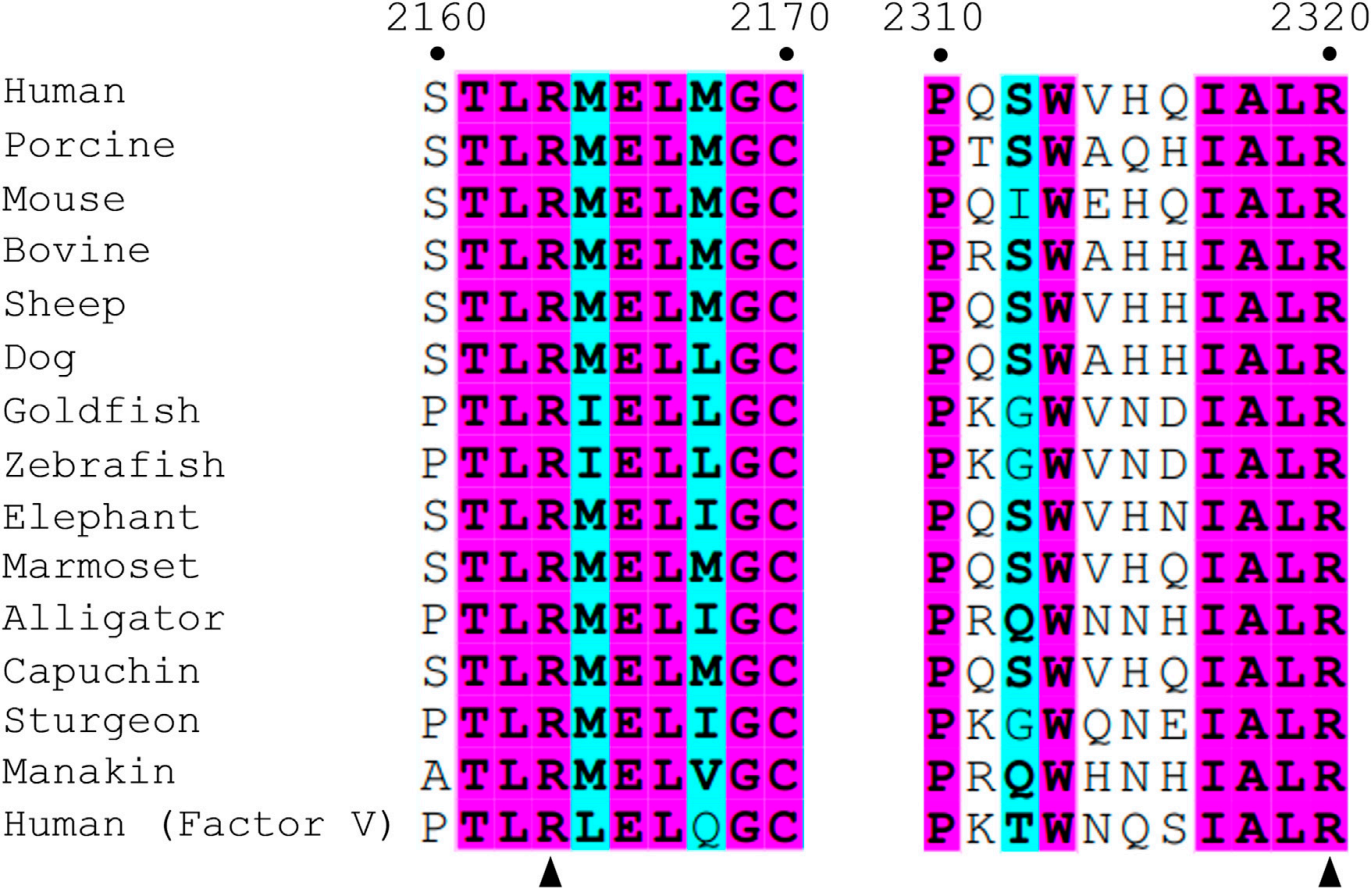
Sequence alignment of fVIII among various species and fV. Numbering is based on human fVIII residues. Similar residues are highlighted in cyan and wholly conserved residues are highlighted in magenta. Arrows point to two conserved arginine residues, R2163 (C1 domain) and R2320 (C2 domain), within the scope of the paper.

Crystal structures of isolated C2 bound to classical antibody inhibitors which disrupt membrane binding have provided unique structural insight into how fVIII docks onto lipid membranes. Structural analysis of C2 bound to BO2C11, a classical antibody inhibitor from a severe hemophilia A patient, supports the model of fVIII platelet association through residues M2199-F2200 and L2251-L2252 as they are completely sequestered in the BO2C11 paratope, potently blocking membrane binding (Figure 1C) (Jacquemin et al., 1998; Spiegel et al., 2001). A ternary complex of C2 bound to antibody inhibitors G99 (non-classical, blocking vWF release) and 3E6 (classical, blocking PS and vWF binding) revealed a unique epitope of C2 postulated to interact with PS headgroups in concert with two hydrophobic loops (Walter et al., 2013). The current model, positioned with the 3E6 epitope in contact with the membrane surface, centers around R2320 and positions residues K2183, D2187, and R2215 in direct contact with PS headgroups while hydrophobic loops extend into the anhydrous interior of lipid membranes (Brison et al., 2015). The CDC Hemophilia A Mutation Project (CHAMP) has identified serine, threonine, tryptophan, and methionine hemophilia A-related missense mutations at R2320, with mutational studies indicating a >95% drop in fVIII activity with select point mutations (Liu et al., 2000). This arginine is highly conserved across species and between the C1 (R2163) and C2 (R2320) domains, illustrating the importance of electrostatic interactions with the lipid membrane surface (Figure 2).

In this study, we demonstrate how two conserved arginine residues, R2163 and R2320 in the C1 and C2 domains, respectively, are essential for stable binding to PS membranes, recapitulating the overall binding affinity of fVIIIa. Along with site-directed mutagenesis to C1, C2, and C1C2 constructs, PS binding was measured by sedimentation *via* ultracentrifugation, ELISA, and biolayer interferometry (BLI). We also present a 1.3 Å crystal structure of isolated C2 bound to OPLS, providing atomic detail into how fVIII recognizes and binds to activated platelet surfaces, and how hemophilia A-associated mutations to the C2 domain may disrupt fVIII binding to lipid membranes. This study further supports the essential coordination of both C domains to facilitate optimal binding and provides evidence demonstrating the involvement of R2163 and R2320 in binding to activated membrane surfaces.

## 2 Materials and methods

### 2.1 Protein expression constructs human factor VIII C1, C2, and C1C2 domains

The gene for human fVIII C2 domain (residues 2171–2332) was subcloned into a pET32a-TEV(+) plasmid (Genscript) using BamHI and XhoI restriction sites with an N-terminal His_6_-thioredoxin tag. Mutants were manufactured using the QuikChange Lightning Site-Directed Mutagenesis Kit (Agilent) and sequenced (Nevada Genomics). Genes for wildtype and mutant human fVIII C1 (C1, residues 2022–2170) and human C1C2 (C1C2, residues 2022–2332) were chemically synthesized by Genscript in a pET32a-TEV(+) plasmid. Bioengineered human/porcine chimeric fVIII variant (ET3i) was a gift from C. Doering (Emory University).

### 2.2 Expression and purification of wildtype and mutant human C2 domain

Wildtype and mutant C2 domains were expressed in SHuffle T7 Express B-strain competent cells (New England Biolabs) (Lobstein et al., 2012). C2 domain mutations (K2187A, D2187A, A2201P, R2215A, K2227A, and R2320S) were selected based on the putative binding model and mutated to alanine or CHAMP-guided hemophilia A associated mutations. Single colonies were grown in LB broth, supplemented with 50 µg/ml ampicillin. Overnight cultures were grown at 30°C until OD_600_ reached a value of 0.6–0.8 and induced with 0.5 mM isopropyl β-D-thiogalactopyranoside (IPTG) at 15°C for 18 h. Protein was purified as previously described, dialyzed in storage buffer (150 mM NaCl, 25 mM Tris-HCl pH 8.0, and 10% (v/v) glycerol), and stored in −80°C at 2–4 mg/ml (Brison et al., 2015).

### 2.3 C1 and C1C2 expression and purification

Wildtype and mutant C1 and C1C2 domain constructs (residues 2022–2170 and 2071–2332, respectively) were expressed in SHuffle T7 K12-strain competent cells and grown in LB media supplemented with 1% anhydrous ethanol, 10 mM MgCl_2_, and 0.4% glycerol (Lobstein et al., 2012; Chhetri et al., 2015). Bacterial cell cultures were shaken until an OD_600_ of 0.4–0.6, rapidly cooled on ice with the addition of 0.5 mM IPTG, and then incubated overnight at 15°C. Expressed cultures were pelleted by centrifugation, lysed and clarified in lysis buffer (20 mM Tris-HCl pH 8.0, 300 mM NaCl, 5 mM imidazole, 10% (v/v) glycerol, 0.1% (v/v) Triton X-100, and 10 mM MgCl_2_) (Brison et al., 2015). Filtered lysate was incubated with buffer-equilibrated TALON resin, washed with 40 column volumes (CV) of lysis buffer supplemented with 10 mM ATP to remove contaminating chaperone proteins (Morales et al., 2019); and eluted with elution buffer [20 mM Tris-HCl pH 8.0, 300 mM NaCl, 500 mM imidazole, and 10% (v/v) glycerol]. Immobilized metal affinity chromatography (IMAC) eluent was diluted with HBS (50 mM HEPES pH 7.4, 300 mM NaCl) and loaded onto a 5 ml HisTrap HP column. A 20 CV wash was performed with HBS containing 20 mM MgCl_2_ and 10 mM ATP followed by a 10 CV HBS wash with 40 mM imidazole. The C1 and C1C2 domain protein constructs were then eluted by a 40–500 mM imidazole gradient, buffer exchanged into low pH HBS (20 mM HEPES pH 6.8, 150 mM NaCl, 10% glycerol) by size-exclusion chromatography (Superdex 75 Increase 10/300; Cytiva), and stored at −80°C (0.3 mg/ml). Expression and purification of wildtype porcine C2 was performed as previously described (Brison et al., 2015).

### 2.4 Preparation of unilamellular vesicles

Preparation of unilamellular vesicles was performed as previously reported with the following adjustments (Julkowska et al., 2013). Unilamellular vesicles were formed using 1, 2-dioleoyl-sn-glycero-3-phosphocholine (DOPC) and 1, 2-dioleoyl-sn-glycero-3-phospho-L-serine sodium salt (DOPS) in 100% DOPC and 80% DOPC/20% DOPS molar ratios. Lipids were resuspended to 1 mM in 20 mM HEPES pH 7.4, 50 mM NaCl, and 0.01% NaN_3_ and incubated for 1 h at room temperature with intermittent mixing. The sample was subsequently vortexed and subjected to four freeze-thaw cycles before passing through an Avanti Mini Extruder 11 times and stored at 4°C.

### 2.5 Lipid sedimentation assays

Sedimentation assays were performed by mixing 125 μl of 1 mM unilamellular vesicles with either 0.2 µM (ET3i or C1C2) or 5 µM (C1 or C2) of protein and buffer (20 mM HEPES pH 7.4, 50 mM NaCl, and 0.01% NaN_3_) to a final volume of 300 µl. Binding reactions were incubated at room temperature for 5 min and centrifuged at 168,000 xg for 45 min at 4°C. Unbound proteins in the supernatant were collected by five-fold excess cold acetone precipitation. Lipid and supernatant pellets were analyzed by SDS-PAGE in a 12.5% Tris-glycine polyacrylamide gel. Relative band intensities were compared using Image Lab and a two-way ANOVA (analysis of variance) comparing each mutant to wild-type C2 retention was performed using GraphPad Prism 9.1.2 (GraphPad Software) followed by a Dunnett’s posttest.

### 2.6 PS-binding enzyme linked immunosorbent assays

PS-Binding ELISA assays were performed as previously described with the following modification (Spiegel et al., 2004). ELISA plates were coated with an 80:20 DOPC: DOPS molar ratio at 10 mg/ml dissolved in methanol. Negative control wells were coated with 100% DOPC at 10 mg/ml. Wells were blocked with TBS supplemented with 1% (w/v) BSA and 1:1 serially diluted protein samples were incubated for 90 min. Subsequently, Ni-NTA•HRP was diluted 1:1,500 with TBS/BSA for 30 min. Lastly, 2, 2′-azinobis (3-ethylbenzthiazoline-6-sulfonic acid) (ABTS) was used to detect binding by absorption at 405 nm. Salt gradient ELISA experiments were performed as above in blocking solution containing 50 mM, 100 mM, 150 mM, or 225 mM NaCl concentrations. All incubation times were performed at 37°C with shaking of 75 rpm. Following data collection, binding curves were normalized, and apparent equilibrium binding affinities were analyzed with a 1:1 saturation binding model with GraphPad Prism. Calculation of error was performed by standard deviation from at least three independent experiments.

### 2.7 Lipid nanodisc assembly

Membrane scaffold protein MSP1D1 was purified as previously described and lipid nanodisc formation was modified as follows (Li et al., 2019). DOPC and DOPS in chloroform were mixed at 100:0 or 80:20 DOPC:DOPS molar ratios and dried under argon gas. Lipids were solubilized in TBS (20 mM Tris-HCl pH 8, 100 mM NaCl, 0.5 mM EDTA) supplemented with 100 mM sodium cholate to a final concentration of 50 mM under 45°C running tap water and vortexed until clear. TEV-cleaved MSP1D1 was incubated with solubilized lipids at a 1:200 protein to lipid molar ratio for 1 h at 37°C at a final concentration of 6 mM total lipids and 20 mM cholate. To initiate assembly of lipid nanodiscs, activated Bio-Beads (BioRad) were added and the sample was allowed to rotate overnight at 4°C. The filtered sample was injected onto an Superdex 200 column 10/300 (GE Healthcare) for final purification.

### 2.8 Binding of factor VIII constructs to lipid nanodiscs

Binding interactions of C1C2 and mutants to lipid nanodiscs were measured on a ForteBio BLItz bio-layer interferometer at 24°C with shaking at 1,800 rpm. Anti-Penta His tips (ForteBio) were pre-hydrated in HBS and loaded with His_6_-tagged C1C2 over 60 s. Lipid nanodiscs were serially diluted 1:1 in HBS and associated to immobilized C1C2 domains over three independent trials. Lipid nanodiscs formed with 100% DOPC at a 1:200 ratio was measured for background subtraction.

Raw data were exported to Microsoft Excel and step corrected for total C domain binding (loading) and detection (association). Loading was determined by the change in wavelength shift from 30 to 90 s minus the change in wavelength shift from 90 to 120 s. The loading was utilized to generate a detection-multiplication factor to account for differences in C domain loading onto the BLI biosensor. Average-adjusted detection shifts were plotted against lipid nanodisc concentration and normalized using GraphPad Prism (GraphPad Software). *K*_D_ values were determined by nonlinear least-squares regression assuming 1:1 stoichiometry. Association and dissociation curves were adjusted using the detection-multiplication factor and plotted against time in seconds. Raw detection values were averaged, multiplied, normalized to the highest detection value, and plotted with GraphPad Prism. Global binding analysis was generated using a kinetic nonlinear regression equation from GraphPad Prism.

### 2.9 Crystallization and structural determination of porcine C2 domain/O-phosphatidyl-L-serine complex

Diffraction quality crystals of porcine C2 were grown by hanging drop vapor diffusion as previously published (Brison et al., 2015). Crystals were soaked in 5 mM OPLS and cryoprotected with a 1:1 addition of 0.1 M CHES pH 10.4, 0.1 M magnesium acetate, and 30% (v/v) glycerol. X-ray diffraction data was collected to 1.3 Å resolution at the Advanced Light Source (ALS) Berkeley Center for Structural Biology (BCSB) 5.0.1 beamline. Data collection and processing were performed with Adxv, XDS and CCP4 (Winn et al., 2011; Porebski et al., 2013). Phasing was accomplished using PHASER-MR with the previously determined 1.7 Å porcine C2 structure (PDB ID: 4MO3) (Adams et al., 2010; Brison et al., 2015). Model building and refinement were performed with WinCoot and PHENIX, respectively (Adams et al., 2010; Emsley et al., 2010). The soluble PS headgroup, OPLS, was modeled within positive density (Supplementary Figure S1) using PHENIX LigandFit and further refined in real space. The correlation coefficient was determined to be 0.748, supporting proper placement of the ligand. All structure figures and structural alignments were generated with PyMOL Molecular Graphics System, Version 2.0 (Schrödinger).

## 3 Results

### 3.1 Isolated C domains bind to lipids with similar affinity

**FIGURE 3:**
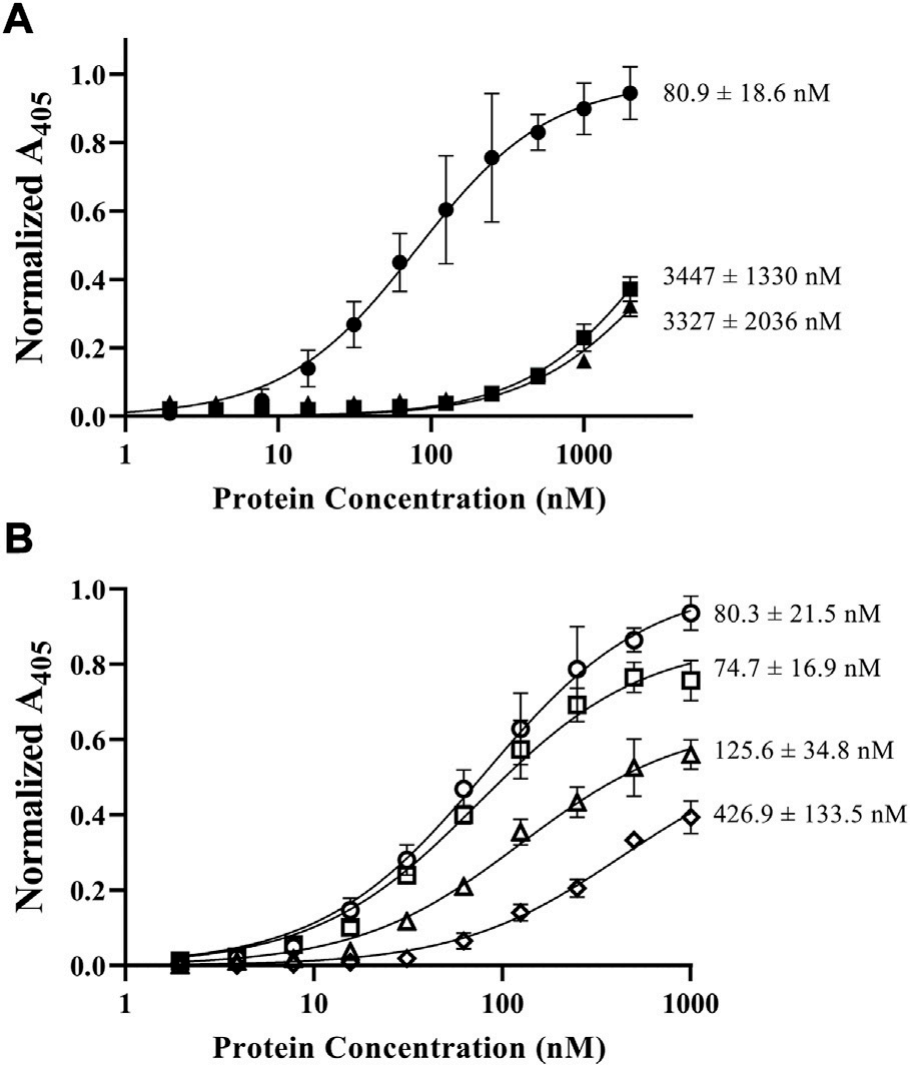
ELISA results of fVIII C domain constructs binding to phosphatidylserine-coated plates. Dissociation constants (*K*_D_) are denoted at the end of each curve. **(A)** Comparison of C1C2 (closed circle), C1 (closed square), and C2 (closed triangle) to lipid unilamellular vesicles (80% PC/20% PS) in TBS. **(B)** Comparison of fVIII C1C2 construct affinity to phospholipids (80% PC/20% PS) in 50 mM (open circle), 100 mM (open square), 150 mM (open triangle), and 225 mM (open diamond) NaCl.

The importance of both C domains in the ability for fVIII to bind lipid membranes has been well established (Scandella et al., 1995; Gilbert et al., 2002; Hsu et al., 2008; Meems et al., 2009; Liu et al., 2010). To confirm the affinity of the isolated domains to phospholipids, we employed an ELISA method using the His_6_-tagged C1, C2, and C1C2 domains to phospholipid-treated plates. Triplicate binding to 80:20 DOPC:DOPS coated plates revealed nanomolar binding affinities for C1C2 with an apparent *K*_*D*_ of 81 ± 19 nM while isolated C1 and C2 domains had similar affinities, measuring 3.4 ± 1.3 µM and 3.3 ± 2.0 µM, respectively (Figure 3A). Additionally, to confirm the role of electrostatic interactions between the C domains and phospholipid surfaces, we measured binding affinities of the C1C2 domain as a function of NaCl concentration (Figure 3B). Binding measurements demonstrated an inverse relationship between NaCl concentration and binding affinity, with NaCl concentrations of 50 mM, 100 mM, 150 mM, and 225 mM associated with apparent *K*_*D*_ values of 80.3 ± 21.5 nM, 74.7 ± 16.9 nM, 125.6 ± 34.8 nM, and 426.9 ± 133.5 nM, respectively. To determine whether high salt concentrations could disrupt C1C2 binding to phospholipid surfaces by inducing the formation of higher order C1C2 oligomers, size-exclusion chromatography was performed on purified wildtype C1C2 at 225 mM NaCl. C1C2 eluted with a single retention volume consistent with a monomeric species (Supplementary Figure S2).

### 3.2 C2 domain mutations decrease binding for PS-containing lipid vesicles

**FIGURE 4:**
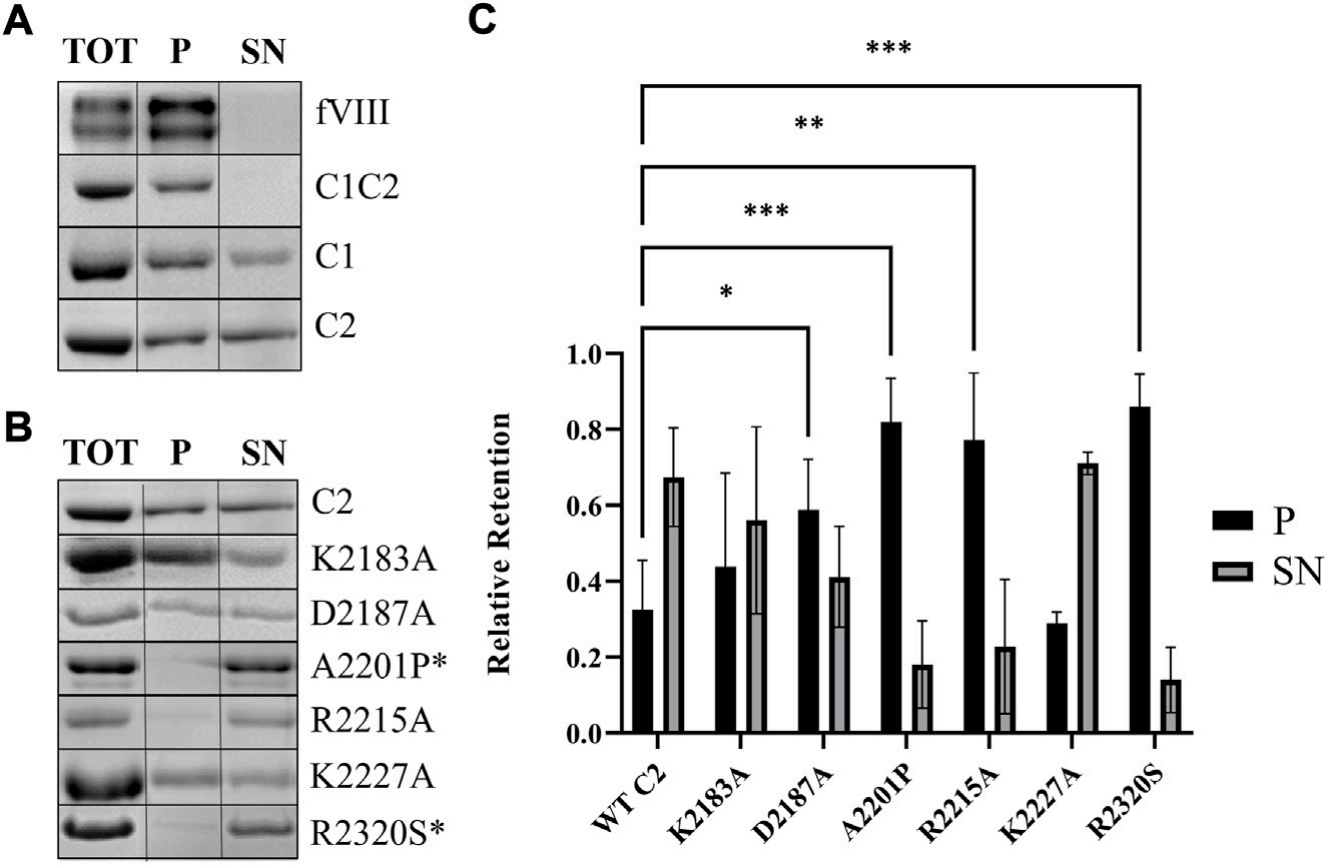
Association of fVIII C2 domains to unilamellular vesicles. (A) SDS-PAGE gel from sedimentation assay of fVIII (heavy and light chains), C1C2, C1, and C2 binding to lipid vesicles (80% PC/20% PS). **(B)** SDS-PAGE gel from sedimentation assays of wildtype and mutant C2 with lipid vesicles (80% PC/20% PS). Aliquots were taken from supernatant (SN) and pellet (P) to determine protein presence. Total (TOT) corresponds to the SN of 100% DOPC vesicles. *Hemophilia A-associated mutations. **(C)** Quantitation of liposome binding assay from Figure 3B from supernatant (black) and pellet (gray). A two-way ANOVA comparing each mutant to wildtype C2 was performed using GraphPad Prism 9.1.2, with three or more independent trials, and Dunnett’s posttest. *p < 0.05, ***p < 0.001, ****p < 0.0001.

Sedimentation assays with 80:20 DOPC:DOPS lipid vesicles were performed to generate a solution-based binding assay that mimics activated platelets. Mature fVIII and wildtype C1C2 bound with >95% retention to DOPC:DOPS-containing vesicles (Figure 4A). When incubated at concentrations near the apparent *K*_D_ (5 µM), isolated C1 and C2 domains bound with approximately 70% retention. To ensure sedimentation was not due to aggregation or insolubility, we observed no binding with vesicles composed of 100% DOPC. We then investigated the working model for fVIII C2 domain membrane association by alanine-scanning mutagenesis and hemophilia A-associated mutants from the CHAMP database using the isolated C2 domain (Liu et al., 2000; Walter et al., 2013; Brison et al., 2015). Mutants K2183A and K2227A resulted in similar retention as wildtype C2 to DOPC:DOPS vesicles (Figure 4B) while binding retention of the D2187A mutant was reduced to 41%. The PS membrane binding model mutants, A2201P, R2215A, and R2320S, also resulted in substantial decreases in retention, binding to DOPC:DOPS vesicles at 18%, 23%, and 14%, respectively (Figures 4B,C). To corroborate the differences in retention of the R2215A and R2320S, ELISA experiments were completed under similar conditions, with both isolated R2215A and R2320S having apparent *K*_D_ values greater than 1 mM (Supplementary Figure S3).

### 3.3 Dual mutations R2163S and R2320S abolish membrane binding

**FIGURE 5:**
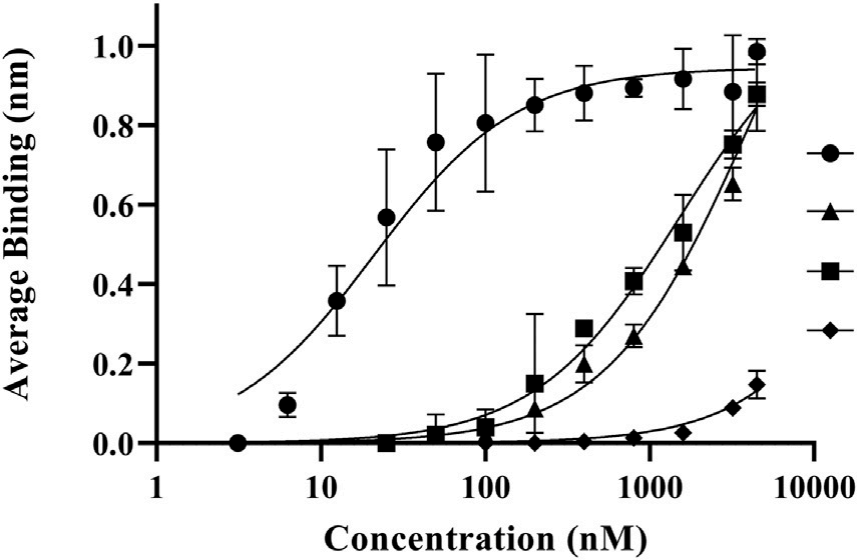
Biolayer interferometry binding measurements of fVIII C1C2 domain mutations to lipid nanodiscs. Average association binding for bound C1C2 wildtype (closed circle), R2163S (closed triangle), R2320S (closed square), and R2163S/R2320S (closed diamond) with lipid nanodiscs (80% PC/20% PS). A baseline was established from 100% DOPC nanodiscs.

To understand the association and dissociation rates of C1 and C2 domain mutations to PS-containing lipid membranes, we measured binding rates and affinities with lipid nanodiscs. Binding kinetics and affinities were subsequently measured between lipid nanodiscs and immobilized wildtype C1C2, R2163S, R2320S, and R2163S/R2320S (Figure 5). Immobilized wildtype C1C2 displayed the highest affinity for nanodiscs with an apparent *K*_*D*_ of 8.9 ± 7.2 nM (Table 1). The apparent affinities of R2163S and R2320S were 160-fold and 326-fold lower, respectively, due to rapid dissociation rates (0.055 ± 0.0042 and 0.24 ± 0.012 min^−1^, respectively) while the double mutant R2163S/R2320S had no detectable affinity (Supplementary Figure S4).

**TABLE 1:** Apparent binding kinetics and affinities of fVIII C1C2 mutations with lipid nanodiscs.

	k_on_ (× 10^4^ M^−1^ s^−1^)	k_off_ (× 10^−3^ s^−1^)	*K*_D_ (μM)
C1C2	14.7 ± 0.16	1.31 ± 0.56	0.0089 ± 0.0072
R2163S	3.81 ± 0.21	54.8 ± 4.2	1.4 ± 0.031
R2320S	8.23 ± 0.38	241 ± 12	2.9 ± 0.015
R2163S/R2320S	0.0010 ± 0.0006	89.7 ± 9.3	>1,000

Data represent the average of three or more independent experiments with a 95% confidence interval. C1C2 wildtype, R2163S, R2320S, and R2163S/R2320S were loaded onto a Penta-HIS BLI sensor and associated to lipid nanodiscs. *k*_on_, rate of association; *k*_off_, rate of dissociation; *K*_D_, dissociation constant (*k*_off_/*k*_on_).

### 3.4 X-ray crystal structure of porcine C2 bound to O-phosphatidyl-L-serine

**FIGURE 6:**
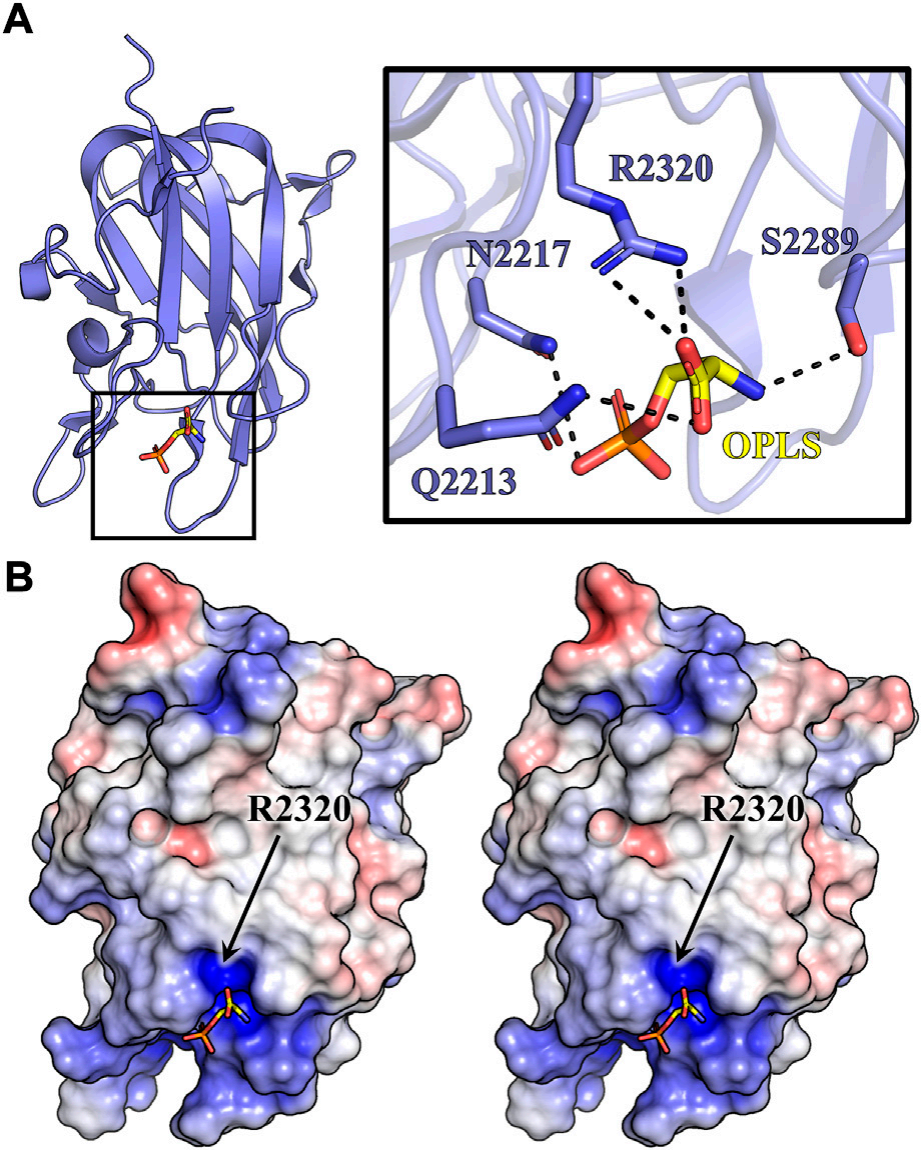
Crystal structure of C2:OPLS. C2 crystals were grown *via* hanging drop vapor diffusion and soaked in 10 mM OPLS. **(A)** Crystal structure of porcine C2 bound to OPLS. Inset depicts OPLS (yellow) forming multiple noncovalent contacts (dashed lines) with C2 residues (slate). **(B)** Stereo view of the electrostatic surface map of the C2:OPLS crystal structure (red: negative charge, blue: positive charge, white: neutral). OPLS (sticks) is situated within the most basic cleft on the C2 domain.

The structure of C2:OPLS complex was determined to 1.3 Å resolution and refined to an R_work_ and R_free_ of 13.5% and 15.7%, respectively (Table 2). Structural alignment to the OPLS-free porcine C2 crystal structure (PDB ID: 4MO3) indicates a shared conformation with an all-atom RMSD of 0.052 Å^2^. Real space modeling of the OPLS ligand positions the carboxy end of OPLS 3.2 Å and 3.0 Å from the R2320 guanidino group (Figure 6A). The carbon backbone of OPLS extends away from the core of the C2 domain, adjacent to the solvent exposed hydrophobic loops previously demonstrated to be an important determinant for PS membrane binding (Pratt et al., 1999; Gilbert et al., 2002). Within the structure, a 3.3 Å hydrogen bond is observed between the S2289 hydroxyl group and the amino moiety of OPLS while N2217 forms two amide nitrogen contacts to the phosphate group at 3.1 and 3.4 Å. A single interaction exists between the Q2213 amide-nitrogen and both carboxyl oxygens of OPLS with additional polar interactions between amide side chains of Q2213 and N2217 at 3.7 Å. Analysis of surface electrostatics illustrates how OPLS binds to a positively charged pocket in the C2 domain centered on residue R2320 (Figure 6B). Visible deviations between 4MO3 and the structure reported herein (PDB ID: 7S0P) concern the hydrophobic loops 2250–2252 and 2197–2200. Although the structure is high resolution, poor electron density is observed for the two β-hairpin loops, indicating high dynamic character. Two alternative conformations for the 2197–2200 loop were resolved in the final model to account for the flexible nature of these hydrophobic loops.

**TABLE 2:** X-ray data collection and model refinement statistics.


X-ray data statistics	
Wavelength (Å)	1.00 Å
Resolution range (Å)	44.53–1.3 (1.346–1.3)
Space group	I 2 2 2
Unit cell (Å)	a = 49.1, b = 68.3, c = 106.0, α = β = γ = 90°
Total reflections	85,226 (6,517)
Unique reflections	42,675 (3,297)
Completeness (%)	96.54 (75.65)
Mean I/sigma (I)	18.77 (2.33)
R_pim_	0.01354 (0.2791)
CC1/2	0.998 (0.832)
Model refinement statistics	
R_factor_	0.1354 (0.2106)
R_free_	0.1570 (0.2308)
Number of Atoms	1,544
Protein	1,362
Water	182
Protein residues	157
RMS bonds (Å)	0.007
RMS angles (°)	1.07
Ramachandran favored (%)	93.55
Ramachandran outliers (%)	0.00
Rotamer outliers (%)	1.95
Average B-factor (Å^2^)	21.53
Protein	20.25
Solvent	30.17
Ligand	132.43

Note: statistics for the highest-resolution shell are shown in parentheses.

## 4 Discussion

The fVIII C2 domain has been shown to play a critical role in platelet binding, with more recent data suggesting the C1 domain contributes substantially to membrane binding (Hsu et al., 2008; Meems et al., 2009; Wakabayashi et al., 2010; Ebberink et al., 2017). Electrostatic contributions from both C domains in binding to negatively charged lipid membranes, however, remains unclear. Several studies have demonstrated that fVIII interacts specifically with OPLS, with over 95% loss of fVIII activity in the presence of the D-isomer of PS (Gilbert and Drinkwater, 1993; Purohit et al., 2005; Miclea et al., 2007), suggesting fVIII contains at least one stereospecific binding site for OPLS among the membrane binding C domains. Given the structural homology and sequence identity between C1 and C2 (Figures 1A, 2), it is plausible that a conserved membrane-binding motif exists within each domain. In the present study, we provide biochemical and X-ray crystallographic data demonstrating a conserved fVIII arginine residue in the C domains that directly binds to PS-containing lipid membranes through electrostatic interactions.

### 4.1 Electrostatics are an essential component of PS binding

We measured binding of fVIII C1, C2, and C1C2 to DOPC:DOPS-coated ELISA plates in physiologically relevant conditions. Experiments with soluble C domain constructs measured weak affinities of isolated C1 (3.4 μM) and C2 (3.3 μM) domains, 10-fold higher than previously reported (Novakovic et al., 2011). The relationship between NaCl and fVIII C2 domain binding has been previously documented, where inhibition of binding was observed for isolated C2 in 150 mM NaCl but retained for fVIII (Novakovic et al., 2011). We report on a similar relationship for C1C2, as an increase of NaCl concentration up to 225 mM reduced the affinity five-fold. Taken together, the observed weak affinity can be attributed to an increase in salt concentration at which binding experiments were performed.

### 4.2 Abrogation of PS binding confirms C domain avidity

*In vitro* fVIII binding to membrane surfaces that mimic activated platelets occurs with high affinity to lipid vesicles (Saenko et al., 1998; Saenko et al., 1999). The binding measurements performed in this study with BLI demonstrate that isolated C1C2 binds to PS-containing lipid nanodiscs with a nanomolar affinity (9 nM), consistent with previous literature values for fVIII, fVIIIa, and fVIII light chain with lipid vesicles (Saenko et al., 1998; Saenko et al., 1999). To our knowledge, this is the first report of quantitative binding measurements for the isolated C1C2 domain, which supports a model wherein the C domains are primarily responsible for PS membrane association. Isolated C domains have been reported to have 100-fold lower affinity for phospholipid membranes compared to fVIII, suggesting synergistic binding through avidity occurs for the tandem C domains (Takeshima et al., 2003). A 160-fold and 320-fold reduction in binding affinity to lipid nanodiscs was observed in C1C2 R2163S and R2320S constructs, respectively (Figure 4). The resultant equilibrium dissociation constants are similar to isolated wildtype C1 and C2, indicating minimal C domain PS binding capability when either R2163 or R2320 is modified. Correspondingly, the C1C2 R2163S/R2320S double mutant abrogated binding. Unlike solvent exposed basic residues K2183, R2222, K2227, and K2249, which display negligible effects on phospholipid binding when mutated, R2320 is partially buried within the β-barrel core of the protein and oriented in such a way that interaction with phospholipids can likely only occur if the hydrophobic spikes are imbedded into the lipid membrane (Liu et al., 2000; Gilbert et al., 2002; Brison et al., 2015). Moderate and severe hemophilia A-associated mutations to R2163 and R2320 are bulky in nature and likely disrupt the protein hydrophobic core by altering internal van der Waals packing, as degradation of fVIII prior to secretion is observed (Liu et al., 2000; Gilbert et al., 2002). By pursuing an arginine to serine substitution associated with mild hemophilia A, we successfully expressed and purified the R2163/2320S mutants in the soluble fraction. BLI assays performed with C1C2 mutant constructs demonstrated robust affinity to non-classical and classical inhibitors, indicating preservation of the structural epitopes associated with fIX and phospholipid binding and maintaining a folded, native state (Supplementary Table S1).

### 4.3 R2163 and R2320 exist within a conserved epitope

Our study also presents the first high resolution structure of an isolated domain from a blood coagulation factor bound to a lipid headgroup moiety. The OPLS group is situated between two loops, L2210-G2214 and S2250-S2253, forming noncovalent interactions with Q2213, N2217, S2289, and R2320. In agreement with our structure, previous homology modeling and membrane binding predictions for C1 and C2 indicate residues R2163, R2220, and R2320 form direct electrostatic interactions with phospholipids (Madsen et al., 2015). Sequence alignment of fVIII and the functionally analogous fV highlights three basic conserved residues in the C2 domain (R2209, R2307, and R2320) that are positioned within previously characterized classical or non-classical inhibitor epitopes. Crystallographic contacts between R2307 and the G99 antibody have been previously determined and subsequent mutational data suggests that mutations at R2307 affect vWF binding and destabilize the structure of the C2 domain (Spiegel et al., 2004). Within the 3E6 epitope, R2209 forms a direct interaction with the 3E6 inhibitor while R2320 is observed to be proximal to the 3E6 CDR loops (Walter et al., 2013). Expression of R2209 mutations in fVIII have demonstrated low expression, but retained greater activity than controls, suggesting a non-essential role in phospholipid binding (Gilbert et al., 2002). Taken together, R2320 appears to be the conserved basic residue that is directly associated with phospholipid binding in the C2 domain whilst the R2163 promotes binding within the C1 domain.

### 4.4 Factor VIII modeling based on juxtaposed C domains suggests partial embedment into membranes

Proposed models of C domain lipid binding vary based on the relative spatial conformation of the tandem C domains and the tilt angle of fVIII relative to the membrane surface. Early models of fVa and fVIII orient the C1 domain stacked above the bound C2 domain, with either the A1 or A3 domain in close proximity to the membrane surface (Pellequer et al., 2000; Stoilova-McPhie et al., 2002). An alternate membrane binding model, consistent with X-ray crystallographic data suggesting a conformationally rigid C1 domain and flexible C2 domain, juxtapositions the C1 and C2 domains at equal depths within the lipid membrane (Smith et al., 2020). Recent high-resolution X-ray diffraction data of a bioengineered fVIII variant with two fVIII molecules in the asymmetric unit revealed two distinct conformations of the C2 domain caused by destabilizing inter-domain shifts observed within the C2-A1 interface (Smith et al., 2020). These conformations, which differ by a rotational tilt about an axis centered in the C2 domain hydrophobic core, retain a juxtaposed orientation while offering an “undocked” and “docked” conformation of the C2 domain when initial membrane binding may be constrained by the C1 domain of fVIII (Smith et al., 2020). However, given the binding dependence of both tandem C domains to retain high affinity binding to lipid membranes, single C domain associations are implausible in a physiological context.

**FIGURE 7:**
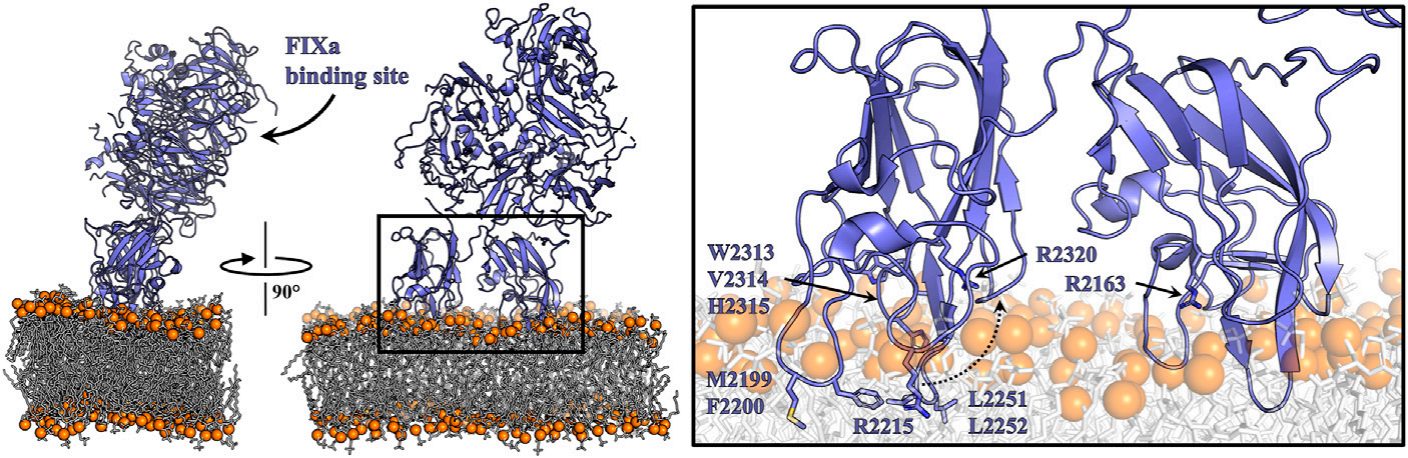
Model of membrane-bound fVIII. Human fVIII (slate) was modeled on a bilipid membrane composed of negatively charged phosphate headgroups (orange) and hydrophobic fatty acid tails (white). Inset depicts fVIII amino acids (sticks) which anchor the cofactor to the lipid membrane. Dashed arrow illustrates the predicted conformational rearrangement to residue R2215 to bind to the negatively charged PS headgroup.

This study demonstrates that the C domains contribute equally to the overall binding affinity of fVIII to PS membrane surfaces. The mutagenesis and binding data from multiple assays, along with the X-ray crystal structure of the isolated C2 domain bound to OPLS, are consistent with an established working model of membrane binding previously reported based on structural studies of B domain-deleted fVIII and anti-C domain antibody inhibitor complexes (Figure 7). This model assumes that each C domain is oriented in a coplanar fashion to interact directly with the PS surface with equivalent binding interfaces, centered on R2163 and R2320. This results in a tilted conformation of fVIII whereby the membrane-facing side of fVIII harbors the fIXa binding region. Upon modeling the tandem C domains in the putative membrane binding orientation, previously reported solvent exposed hydrophobic loops K2092-F2093, M2199-F2200, and L2251-L2255 are positioned below the membrane surface while W2313–H2315 are situated at the membrane surface. The planar membrane sequesters the classical inhibitor epitopes for 3E6 and BO2C11, positioning residues Q2213 and R2215 below the putative membrane surface and K2183, D2187, R2209, and H2211 adjacent to the PS membrane. This putative membrane binding model is further supported by high resolution epitope mapping with site-directed mutagenesis of the C2 domain (Nguyen et al., 2014). Here, alanine scanning demonstrates that the regions described above are critical determinants for binding of classical C2 domain inhibitors, which block PS membrane binding. The position of the completely solvent-exposed R2215 into the interior of the membrane is unlikely, leading to a hypothesis that this flexible residue reaches back toward R2320 and the positively charged binding cleft to form a salt bridge with the phosphate group of OPLS. This rearrangement to R2215 could be accomplished by C2 binding a complete PS molecule incorporating the fatty acid tails. The “non-classical” G99 inhibitor epitope, which does not disrupt PS membrane association of C2 or fVIII, is oriented away from the phospholipid binding interface while the hydrophobic solvent-exposed loops sequestered in the BO2C11 epitope are encased within the anhydrous membrane interior. This coplanar, tilted model of fVIII membrane binding additionally supports previous studies focused on fIXa active site placement and the overall height of the fVIII on lipid membranes at >70 Å and 88 Å, respectively (Mutucumarana et al., 1992; Cheng et al., 2019).

## 5 Conclusion

In this study, the relative contributions of each C domain to PS membrane binding is parsed and quantitatively measured, and the interpretation of these data are consistent with a current working model for fVIII membrane association that involves coplanar binding of both C domain centered on their respective conserved arginine residues, R2163 and R2320. Phospholipid binding assays confirm the synergistic function of the tandem C domains as mutations to R2163 (C1) and R2320 (C2) abrogated binding of each respective domain. A crystal structure of fVIII C2 domain shows R2320 bound to OPLS illustrates the first direct evidence of molecular contacts between the fVIII C domains and a lipid moiety. Utilizing recent high-resolution structures of fVIII alone and in complex with antibody inhibitors led to modeling the fVIII membrane-bound orientation in a tilted state, consistent with previous studies. Our study is consistent with the previously proposed C2 domain phospholipid binding model, which hypothesizes the two solvent-exposed hydrophobic loops (M2199-F2200 and L2251-L2252) that extend into the phospholipid anhydrous interior, positioning the conserved R2320 (and R2163 in C1) at the surface of lipid membranes. Subsequent structural investigations of fVIII bound to soluble lipid bilayers will elucidate hydrophobic interactions between C domain loops and the anhydrous lipid interior and discover additional basic residues involved in electrostatic interactions with phospholipid headgroups.

## Data Availability

The datasets presented in this study can be found in online repositories. The names of the repository/repositories and accession number(s) can be found below: http://www.wwpdb.org/, 7S0P.
