# Supplementary material for: Stable binding to phosphatidylserine-containing membranes requires conserved arginine residues in tandem C domains of blood coagulation factor VIII
Source: Front Mol Biosci. 2022 Oct 26;9:1040106. doi: 10.3389/fmolb.2022.1040106 (PMC9643838; doi:10.3389/fmolb.2022.1040106)
Supplement: Supplementary file 1 [file DataSheet1.docx]

**Supporting information**


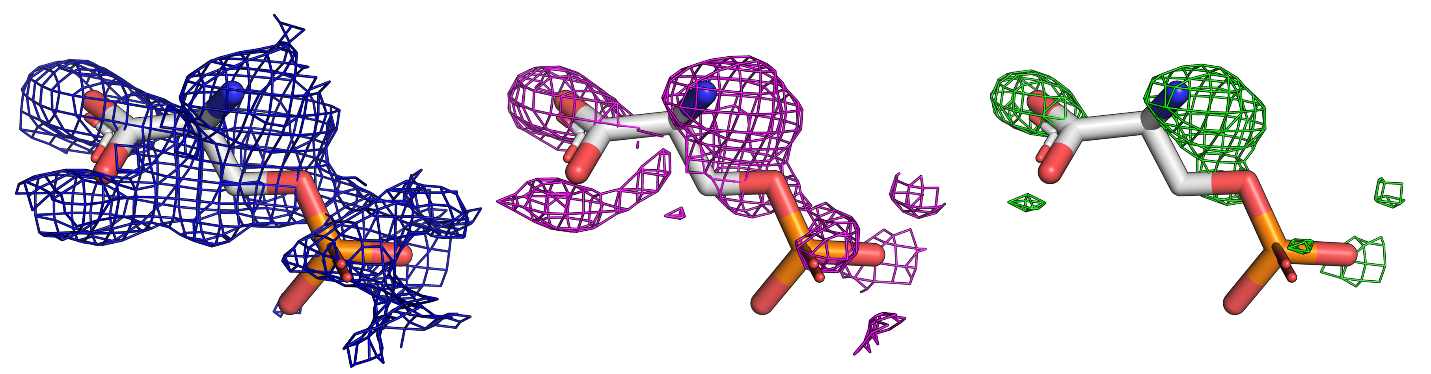


**Figure S1**. **Omit |*F_o_*| – |*F_c_*| density surrounding the OPLS ligand.** Densities were calculated by simulated annealing refinement in PHENIX after removing the OPLS ligand (sticks) and contoured to 1σ (*left, blue*), 2σ (*middle, magenta*), and 3σ (*right, green*).


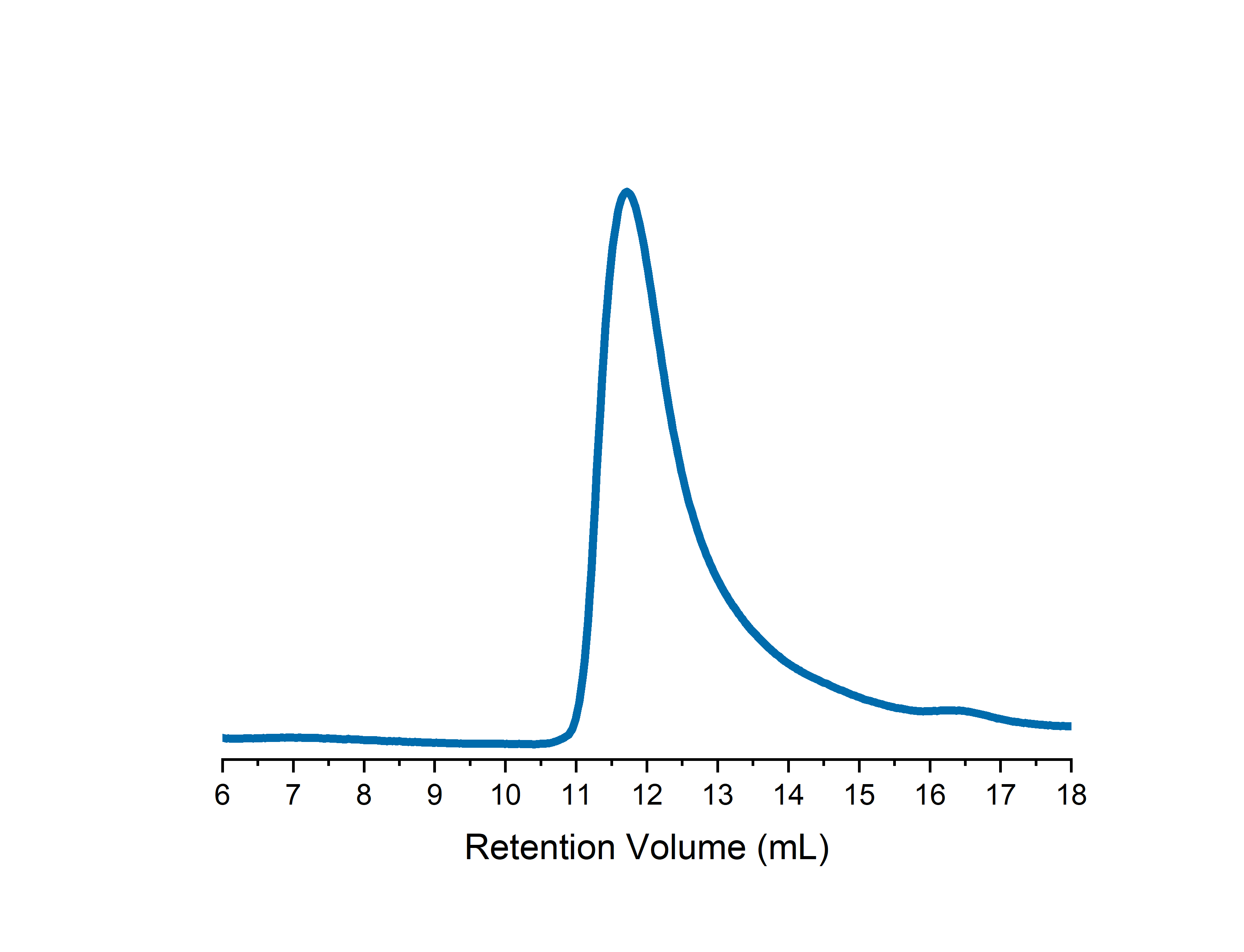


**Figure S2. Size-exclusion chromatography of wildtype C1C2 under 225 mM NaCl.** Wildtype C1C2 was purified by size-exclusion chromatography (Superdex 75) in buffer containing 225 mM NaCl. The retention volume corresponds to monomeric C1C2.


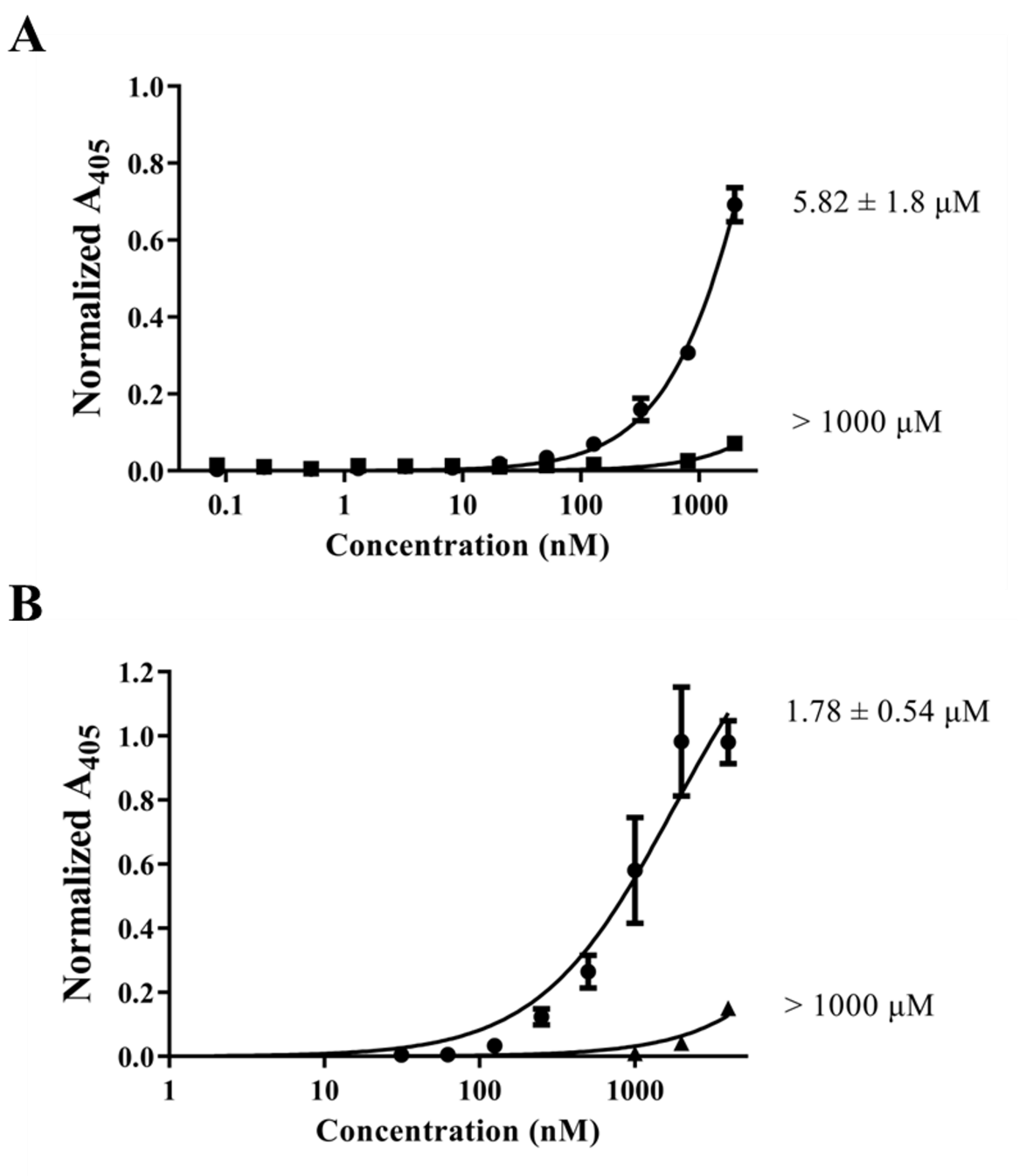


**Figure S3. ELISA results of C2 R2215A and R2320S binding to phosphatidylserine-coated plates.** Dissociation constants (K_D_) are denoted at the end of each curve. **A)** Comparison of wildtype C2 (closed circle) and R2215A (closed square) **B)** Comparison of wildtype C2 (closed circle) and R2320S (closed triangle). Plates were coated with 80:20 DOPC:DOPS. Bound C2 was detected with HRP-conjugated Ni-NTA. HRP was probed with ABTS and color development was measured at 410 nm.


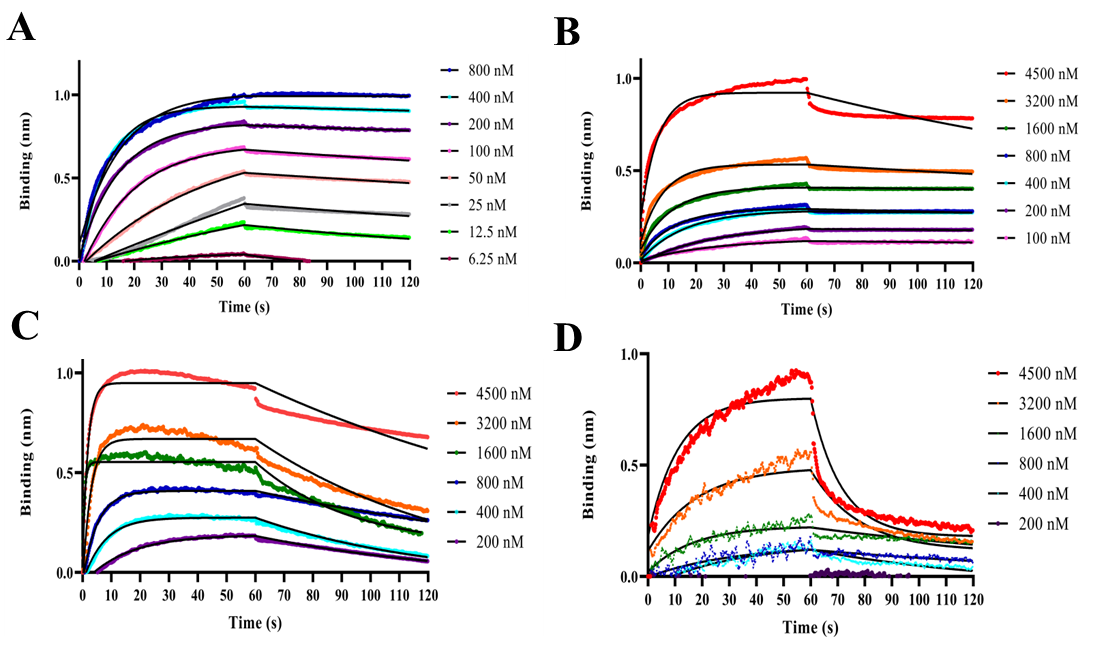


**Figure S4. Association of tandem C domain constructs to lipid nanodiscs.** Association and dissociation binding rates for C1C2 **A)** wildtype, **B)** R2163S, **C)** R2320S, and **D)** R2163S/R2320S with immobilized lipid nanodiscs. Baseline was established from 100% DOPC nanodiscs. C domain loading onto the tips was step-corrected, averaged over three independent trials, normalized using Excel, plotted on GraphPad Prism 9.1.2, and fit using an association and dissociation non-liner regression setting.


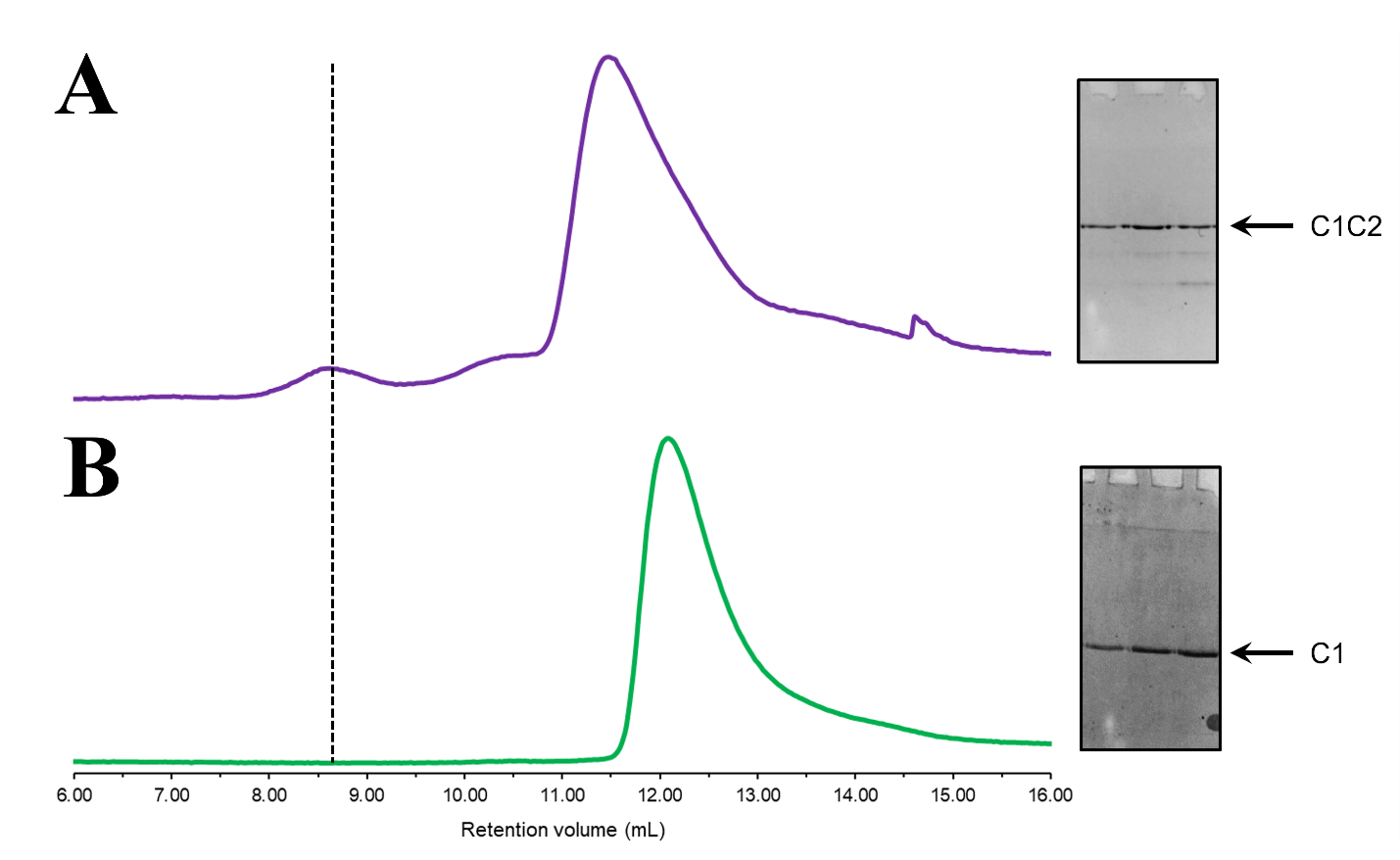


**Figure S5. Size-exclusion chromatography of wildtype human C1C2 and C1.** Chromatograms from size-exclusion chromatography (Superdex 75 10/300) of tagged human C1C2 (A, purple) and C1 (B, green). SDS-PAGE gel analysis of fractions from the main peak support sample purity. Dashed line indicates the void volume of the column.

**Table S1. Dissociation constants for C1 and C2 constructs to inhibitory antibodies.**

|  | **G99**  **K_D_ (nM)** | **2A9**  **K_D_ (nM)** | **3E6**  **K_D_ (nM)** |
| --- | --- | --- | --- |
| fVIII | 13.5 ± 2.9 | 79.3 ± 5.3 | 15.4 ± 3.2 |
| C1 | N/A | 13.6 ± 1.8 | N/A |
| C2 | 50.8 ± 1.9 | N/A | 75.4 ± 1.5 |
| R2320S | 44.0 ± 3.6 | N/A | 57.2 ± 1.8 |
| C1C2 | 38.2 ± 1.0 | 3.19 ± 2.0 | 13.9 ± 1.1 |
| R2163S | 4.34 ± 0.6 | 44.7 ± 2.2 | 15.8 ± 1.7 |
| R2320S | 7.43 ± 0.5 | 65.4 ± 11 | 80.9 ± 1.8 |
| R2163S/R2320S | 5.91 ±1.6 | 56.1 ± 0.5 | 63.0 ± 1.8 |
